# Clinical Outreach Training and Supportive Supervision Quality-of-Care Analysis: Impact of Readiness Factors on Health Worker Competencies in Malaria Case Management in Cameroon, Mali, and Niger

**DOI:** 10.4269/ajtmh.23-0479

**Published:** 2023-12-26

**Authors:** Yves-Marie Bernard, Jehan Ahmed, Jadmin Mostel, Thierno Ba, Annie Coriolan Ciceron, Moses Busiga, Aissata Koné, Beh Kamaté, Fatoumata Sidibé, Chebou Diallo, Alzouma Makayi, Daniel Christian Koko, Arouna Djibrilla, Joël Ateba, Eric Tchinda Meli, Christophe Tchadjeu, Kevin Griffith, Jordan Burns, Lawrence M. Barat

**Affiliations:** ^1^ PMI Impact Malaria, Population Services International, Washington, District of Columbia;; ^2^ Programme National de Lutte contre le Paludisme du Mali, Bamako, Mali;; ^3^ PMI Impact Malaria, Population Services International, Bamako, Mali;; ^4^ Programme National de Lutte contre le Paludisme du Niger, Niamey, Niger;; ^5^ PMI Impact Malaria, Population Services International, Niamey, Niger;; ^6^ Programme National de Lutte contre le Paludisme du Cameroun, Yaoundé, Cameroon;; ^7^ PMI Impact Malaria, Jhpiego, Yaoundé, Cameroon;; ^8^ PMI Impact Malaria, Association Camerounaise pour le Marketing Social, Yaoundé, Cameroon;; ^9^ U.S. President’s Malaria Initiative, United States Agency for International Development, Washington, District of Columbia

## Abstract

Improving the quality of malaria clinical case management in health facilities is key to improving health outcomes in patients. The U.S. President’s Malaria Initiative Impact Malaria Project has supported implementation of the Outreach Training and Supportive Supervision (OTSS) approach in 11 African countries to improve the quality of malaria care in health facilities through the collection and analysis of observation-based data on health facility readiness and health provider competency in malaria case management. We conducted a secondary analysis of longitudinal data collected during routine supervision in Cameroon (April 2021–March 2022), Mali (October 2020–December 2021), and Niger (November 2020–September 2021) using digitized checklists to assess how service readiness affects health worker competencies in managing patients with fever correctly and providing those with confirmed uncomplicated malaria cases with appropriate treatment and referral. Linear or logistic regression analyses were conducted to assess the effect of facility readiness and its components on observed health worker competencies. All countries demonstrated significant associations between health facility readiness and malaria case management competencies. Data from three rounds of OTSS visits in Cameroon, Mali, and Niger showed a statistically significant positive association between greater facility readiness scores (including the availability of commodities, materials, and trained staff) and health worker competency in case management. These findings provide evidence that health worker performance is likely affected by the tools and training available to them. These results reinforce the need for necessary tools and properly trained staff if high-quality malaria case management services are to be delivered at health facilities.

## INTRODUCTION

In the face of numerous challenges to malaria control and elimination efforts, the WHO has reiterated the importance of using a primary health-care approach to strengthen health systems so that high-quality services and interventions can be delivered to and accessed by those in need.^1^ Improving the quality of malaria clinical case management in health facilities through timely and accurate diagnosis and treatment of confirmed cases using artemisinin-based combination therapies (ACTs) is key to improving health outcomes in patients. There is considerable evidence demonstrating the positive impact of training and supportive supervision on improving provider practices.^2^^–^^5^

The U.S. President’s Malaria Initiative (PMI) Impact Malaria Project, launched in 2018, has supported implementation of the Outreach Training and Supportive Supervision (OTSS) approach to improve the quality of malaria care in health facilities in 11 African countries (Cameroon, Côte d’Ivoire, Ghana, Kenya, Madagascar, Malawi, Mali, Niger, Sierra Leone, Tanzania, and Zambia).^6^ This approach, built on the original OTSS approach launched under the PMI Improving Malaria Diagnostics Project (2007–2011) and continued under the PMI MalariaCare Project (2012–2017),^7^^,^^8^ focuses on the continuous improvement of 1) service delivery readiness of health facilities, and 2) competencies of health providers in malaria diagnosis and treatment. Outreach Training and Supportive Supervision supervisors use standard checklists to monitor and track progress by conducting a facility inventory and by observing health workers managing patients directly.

In most countries, OTSS checklist data are collected in the Health Network Quality Improvement System (HNQIS), a digital platform that provides immediate scoring during the OTSS visit. Based on these scores, supervisors provide on-the-job training, coaching, and troubleshooting, and develop action plans to address identified gaps. The OTSS visit results and action plan are followed up between visits to address gaps in the availability of essential malaria drugs and commodities, materials (e.g., job aids and guidelines), and documentation (i.e., registers and reporting forms); and are reinforced during subsequent visits to strengthen continuously health worker competencies to manage malaria cases (including malaria in pregnancy) and perform malaria rapid diagnostic tests (RDTs) properly.

The OTSS readiness checklist (Supplemental Figure 1) collects facility-based information on the availability of malaria commodities, including RDTs and ACTs; materials, including malaria case management guidelines and job aids; documentation (registers and reporting forms); and trained personnel at the facility. The information gathered from the readiness checklist during OTSS visits enables supervisors to track progress on overall facility readiness and each individual checklist component.

The OTSS approach also uses a health provider competency-based checklist—the outpatient department (OPD) checklist (Supplemental Figure 2)—to assess clinicians’ management of suspected malaria patients. Supervisors use this checklist to observe the provider–patient interaction, collecting information on the competency of health workers in welcoming patients, assessing the history of fever, reviewing symptoms, performing a physical examination, requesting appropriate testing (including RDTs), making the correct classification of cases (as nonmalaria, uncomplicated malaria, or severe malaria), providing treatment to patients with positive tests, adhering to negative test results, and providing counseling to patients. This checklist generates data on the overall performance of health workers, as well as for each of the component behaviors.

Previous studies have assessed the impact of facility readiness on the implementation of malaria interventions. Evidence from cross-sectional studies in particular have demonstrated the correlation between facility readiness and quality service delivery.^9^^–^^16^ Similarly, the service availability and readiness assessment methodology has provided information on the availability of health system inputs and their impact on improved health outcomes.^17^ However, there remains a paucity of evidence on the direct impact of these inputs, or facility readiness more generally, on the quality of care provided to febrile patients presenting at health facilities. An analysis of pooled OTSS data from nine countries supported by MalariaCare demonstrated the positive association between the overall performance of health facilities and the competency of health workers.^5^ The study showed a strong positive association between overall performance and key readiness outputs (such as the availability of the most recent malaria case management guidelines and algorithms, and formally trained health workers), but found no significant association between stock-outs of ACTs and health worker competency scores. However, the study did not explore the direct association between the availability of commodities and the specific competencies of health workers in requesting RDTs and prescribing the correct malaria treatment. The study team noted the need for additional evidence on the association between health facility readiness and the quality of malaria case management.

This secondary analysis of OTSS data assesses whether health facility readiness in high-burden countries is associated with health worker competencies in diagnosing and managing patients correctly who are suspected of having malaria, and providing those with uncomplicated malaria with appropriate treatment and referral.

## MATERIALS AND METHODS

A secondary analysis of longitudinal OTSS data collected during routine supervision activities in Cameroon, Mali, and Niger was conducted to assess the association between overall facility readiness and the competency of health workers in managing patients suspected of having malaria, in addition to the association between 1) the availability of trained personnel in facilities and the competency of health workers in assessing fever correctly, including requesting a malaria diagnostic test (microscopy and/or RDTs) and providing the correct treatment for patients diagnosed with malaria; 2) the availability of guidelines, documents, and materials in facilities and the competency of health workers in assessing fever correctly, including requesting malaria tests (microscopy and/or RDTs) and providing the correct treatment for patients diagnosed with malaria; 3) the availability of RDTs and microscopy commodities in facilities and the competency of health workers in requesting a malaria diagnostic test for patients with fever; and 4) the availability of first-line malaria treatment and the competency of health workers in prescribing the correct treatment.

### Study design.

Three countries—Cameroon, Mali, and Niger—were selected for this study because of the similarities in their implementation of OTSS: 1) all started OTSS in 2019; 2) all used the same OTSS tool and checklists, with slight adaptations for country context; and 3) all made similar investments and faced similar challenges in policy development, training, and supply chains for commodities such as RDTs and ACTs. At the time of the study, five rounds of OTSS had been implemented in Cameroon, Mali and Niger since its launch in 2019. A round is defined as a specific period during which a targeted set of facilities receive OTSS visits. The OTSS rounds should happen quarterly in each of the three countries. However, constraints—which are linked primarily to competing government priorities—resulted in each of the countries conducting OTSS rounds every 4 to 6 months. At the launch of this approach, the number of facilities to visit was selected based on PMI Impact Malaria target facilities according to availability of resources. Over time, the initial pool of facilities was increased gradually based on the expansion of Impact Malaria targets.

Three rounds were selected for each country. Rounds 3 through 5 were selected for Cameroon and Mali. For Niger, rounds 2 through 4 were selected because, starting in round 5, OTSS visits shifted to a different set of facilities that had not been visited in previous rounds, making the results from round 5 not comparable to previous rounds. The specific timing of each round is noted in Table 1.

**Table 1:** Number of facilities and observations in final data sets for Cameroon, Mali, and Niger

Country	Round	Timing	No. of facilities	No. of provider observations
Cameroon	3	April–May 2021	200	215
	4	September–October 2021	367	395
	5	February–March 2022	285	285
Mali	3	October–November 2020	235	484
	4	March–April 2021	234	406
	5	November–December 2021	230	396
Niger	2	November–December 2020	93	109
	3	April 2021	66	79
	4	July–September 2021	82	96

Supervisors use digitized checklists that break down the recommended procedures into objective steps (with yes/no questions to indicate whether a step was performed) and are provided periodic refresher training to limit observer bias. Each question in the checklists is assigned a weight of 1 to 3 points based on its importance. In addition, data collected during the observation are reviewed and validated with the observed health provider.

Supervision data collected in the HNQIS (version 1.6.15; Population Services International, Washington, D.C.) and downloaded to a DHIS2 (version 2.36.10.1; University of Oslo, Norway) platform were extracted. Data from each OPD checklist from a round of OTSS visits in a country were matched with the corresponding facility readiness checklist. If there were multiple facility readiness checklist observations for the same facility on the same date, which could have resulted from multiple entries by the supervisor, the observation with the lowest score was retained. All observations for the OPD checklist were retained because countries perform as many as three clinical observations during one OTSS visit. Facilities with data available from both the facility readiness and the OPD checklists within each round were included in the study.

### Measures and variables.

We selected four variables from the OPD checklist (dependent variables) and five variables from the facility readiness checklist (independent variables) for analysis. Variables selected from the OPD checklist included case management competency score, correct classification of malaria, malaria test requested for a child with fever, and malaria treatment provided for a child who tested positive for malaria. Variables selected from the facility readiness checklist included overall readiness score, percentage of facilities with ≥50% of health workers who received classroom training, availability of materials, availability of RDTs, and availability of ACTs.

A competent health worker is defined as one who achieved an overall competency score of 90% on the OPD checklist, which is calculated using all sections of the checklist, including patient assessment, diagnosis, classification, and correct treatment and referral based on RDT result. The case management competency score data were retained as a continuous variable.

Facility readiness is defined as a health facility that achieved an overall readiness score of 90% on the facility readiness checklist, which is calculated using all sections of the checklist, including the availability of malaria commodities, materials, documentation, and ≥50% of personnel receiving classroom training. Our study looked at the availability of two commodities—RDTs and ACTs—as binary variables, where 0 = no commodity available and 1 = the commodity was available. The availability of ACTs was defined by the availability of ACT for all age groups across each country. (In Cameroon, a minimum 1-month supply was required.) In instances where ACTs were not available for a certain age group, ACTs were considered not to be available. Availability of materials is defined as a facility that scores ≥90% in having national malaria guidelines and recommended job aids, as well as necessary malaria registers and reporting forms, per the national guidelines. Our study retained the availability of materials variable as a continuous variable. Facilities with the necessary personnel is defined as those with ≥50% of their personnel (all categories) having received classroom training in malaria case management during the past 2 years.

### Analyses.

The study team first calculated for each country the average health facility readiness and health worker competency scores for all facilities and the proportion that met the 90% threshold score for health facility readiness and health worker competency in case management. In addition, for health facility readiness, the proportion of health facilities that have ≥50% of trained health workers, RDTs available, and ACTs available was calculated, as was the average score of health facilities that have the necessary materials (sum of availability of materials scores divided by the number of facilities). For health worker competency, the proportion of the health workers that classify malaria correctly, request a malaria test, and provide the correct treatment was generated.

Linear regressions assessed the percentage point change in case management competency score (dependent variable) with each 10% increase in overall readiness score (independent variable), the percentage point change in case management competency score (dependent variable) with each 10% increase in materials availability (independent variable), and the percentage point change in case management competency score (dependent variable) when facilities have ≥50% of trained personnel (independent variable).

Logistic regressions were conducted to assess the odds of the health worker providing correct treatment to patients (dependent variable) with the availability of ACTs (independent variable), and the odds of the health worker requesting RDT or microscopy (dependent variable) with the availability of RDT commodities (independent variable). Statistical regressions were deemed significant at *P* <0.05.

### Data management.

Location data such as GPS points were not included in extracted data sets. Facility names were included in the data sets but were not used for the analysis. Analyses and results were only presented at the country level, with aggregate data to ensure results did not contain any personally identifiable information and could not be traced back to a particular individual, facility, or geographic area in-country. All data were stored in a restricted access folder.

### Data validation.

The research team held a validation meeting with PMI Impact Malaria country teams in November 2022 to review preliminary findings for their respective country. During the validation meeting, the research team presented and discussed the findings and relevant contextual information.

## RESULTS

The final data sets used for analysis included a total of 895 observations for Cameroon, 1,286 observations for Mali, and 284 observations for Niger. Details on the number of observations and facilities for each round and country are outlined in Table 1.

**Figure 1.**
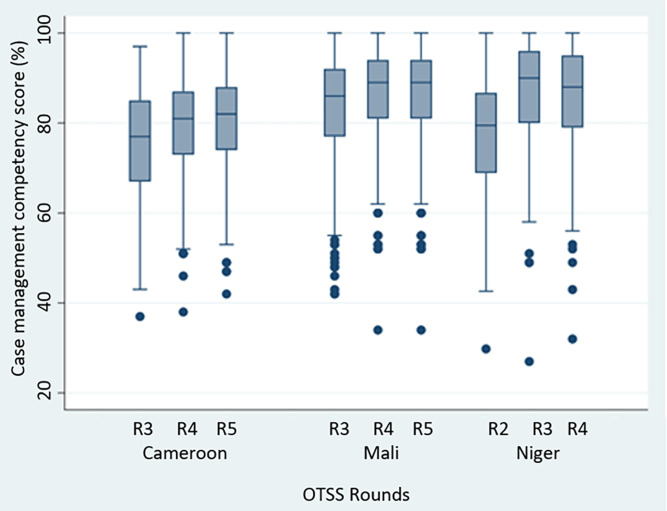
Distribution of case management competency scores by country and round. Cameroon data are from April 2021 to March 2022. Mali data are from October 2020 to December 2021. Niger data are from November 2020 to September 2021. OTSS = Outreach Training and Supportive Supervision; R = round.

Table 2 presents trends in scoring over successive OTSS rounds for the variables chosen for the linear or logistic regression analyses for Cameroon, Mali, and Niger. In the three countries, health workers demonstrated an improvement in case management competency scores through the three rounds analyzed (Figure 1). The percentage of health facilities that achieved a ≥90% overall facility readiness score increased with subsequent OTSS visits across all countries. In Cameroon, the percentage of health facilities that met this 90% threshold increased from 7.9% in round 3 to 60.4% in round 5. In Mali, the percentage that met the threshold increased from 33.1% in round 3 to 41.7% in round 5. Niger also showed an increase in the percentage that met the threshold, from 5.7% in round 2 to 29.2% in round 4. In most instances, commodity and material availability, correct classification of malaria, requesting a malaria test, and providing correct treatment to patients remained high (reaching ≥80%) in each country across all rounds of OTSS visits analyzed.

**Table 2:** Health facility readiness and health worker competency for Cameroon, Mali, and Niger across three rounds of OTSS

Descriptive statistic	Cameroon, *n *(%)			Mali, *n *(%)			Niger, *n *(%)		
	R3	R4	R5	R3	R4	R5	R2	R3	R4
Health facility readiness									
Health facilities that have a 90% score for overall readiness	215 (7.9)	395 (21.0)	285 (60.4)	480 (33.1)	384 (40.6)	374 (41.7)	106 (5.7)	79 (31.6)	96 (29.2)
Health facilities that have ≥50% of trained health workers	215 (36.3)	395 (55.7)	285 (91.2)	482 (41.9)	384 (37.5)	374 (38.5)	106 (27.4)	79 (32.9)	96 (51.0)
Average score of health facilities that have the necessary materials	215 (79.3)	395 (89.5)	283 (77.5)	480 (80.9)	384 (84.7)	374 (86.9)	106 (89.4)	79 (93.0)	96 (94.0)
Health facilities that have RDTs available	215 (96.3)	395 (90.1)	285 (93.7)	482 (93.4)	384 (98.2)	374 (99.2)	106 (99.1)	79 (92.4)	96 (92.7)
Health facilities that have ACTs*	215 (77.7)	395 (53.4)	211 (97.2)	480 (50.6)	384 (69.5)	272 (98.2)	106 (81.1)	79 (91.1)	87 (95.4)
Health worker competency									
Health workers who have a 90% score for competency	211 (10.4)	394 (16.6)	285 (18.9)	475 (37.9)	396 (46.0)	386 (45.6)	98 (16.3)	79 (51.9)	96 (42.7)
Health workers who classified malaria correctly	101 (97.0)	311 (96.8)	153 (93.5)	282 (93.6)	104 (98.1)	94 (97.9)	89 (100.0)	60 (98.3)	87 (100.0)
Health workers who requested a malaria test^†^	215 (97.2)	394 (97.5)	285 (98.6)	478 (94.4)	396 (95.0)	386 (94.8)	109 (100.0)	79 (97.5)	96 (97.9)
Health workers who provided the correct treatment^‡^	87 (83.9)^§^	255 (94.9)^ǁ^	120 (98.3)^ǁ^	183 (97.3)^ǁ^	76 (100.0)^ǁ^	74 (100.0)^ǁ^	84 (98.8)^ǁ^	56 (100.0)^ǁ^	75 (100.0)^ǁ^

ACT = artemisinin-based combination therapy; R = round; RDT = rapid diagnostic test.

* The ACT variable was calculated in a binary way (0/1) as 1 = all age groups had ACTs available and 0 = at least one age group did not have ACTs available.

^†^ A malaria test was defined as an RDT or microscopy. Because of indicator wording, RDT and microscopy could not be disaggregated.

^‡^ The variable for providing the right treatment was split into two categories: for pregnant women or for nonpregnant women. Except for Cameroon round 3, all data for nonpregnant women were used.

^§^ Cameroon round 3 had data for pregnant women only; there were no data for nonpregnant women.

^^ǁ^^ Nonpregnant women.

Similarly, there were improvements between the first and last assessed rounds for each country for the percentage of health worker observations that met the 90% score for case management competency across the three countries: 10.4% in round 3 to 18.9% in round 5 for Cameroon, 37.9% in round 3 to 45.6% in round 5 for Mali, and 16.3% in round 2 to 42.7% in round 4 in Niger.

### Statistical regression analysis.

All countries demonstrated significant associations between overall health facility readiness and case management competency (Table 3). In Cameroon and Mali, all three rounds of OTSS indicated this positive association. In Niger, only round 3 of OTSS indicated a significant relationship between overall readiness and case management competency.

**Table 3:** Association between provider competency and health facility readiness in Cameroon, Mali, and Niger over three rounds of OTSS

Descriptive statistic	Cameroon			Mali			Niger		
	R3, % (95% CI)	R4, % (95% CI)	R5, % (95% CI)	R3, % (95% CI)	R4, % (95% CI)	R5, % (95% CI)	R2, % (95% CI)	R3, % (95% CI)	R4, % (95% CI)
Percentage change in case management competency score (*y*) with each 10% increase in overall readiness score (*x*)	**3.7** (1.9–5.4)*	**4.8** (3.7–5.9)*	**2.2** (1.3–3.2)*	**3.0** (1.8–4.3)*	**1.9** (0.7–3.1)^†^	**2.9** (1.6–4.4)*	3.8 (–0.4 to 8.0)	**5.3** (1.5–9.1)^†^	2.5 (–0.8 to 5.8)
Percentage change in case management competency score (*y*) with each 10% increase in materials available (*x*)	**2.1** (1.3–2.9)*	**2.4** (1.8–3.0)*	0.3 (–0.1 to 0.6)	0.1 (–0.3 to 0.5)	0.1 (–0.4 to 0.5)	0.3 (–0.2 to 0.8)	**2.5** (0.1–4.9)^‡^	2.2 (–0.9 to 5.4)	0.7 (–2.0 to 3.3)
Percentage change in case management competency score (*y*) when facilities have ≥50% trained personnel (*x*)	2.4 (–1.0 to 5.9)	0.6 (–1.6 to 2.7)	4.3 (–0.1 to 8.8)	**5.4** (3.3–7.6)*	**3.1** (1.0–5.3)^†^	**3.4** (1.2–5.6)^†^	–2.6 (–9.2 to 4.1)	3.2 (–3.4 to 9.8)	–0.9 (–6.7 to 4.9)

* *P* <0.001.

^†^ *P* <0.01.

^‡^ *P* <0.05.

Values in bold type are statistically significant.

Cameroon and Niger’s data indicated a significant association between the availability of materials and case management competency (Table 3). There was no significant association in Mali. In Mali, there was a significant association between prior health worker training and case management competency. Similar associations were not identified in Cameroon and Niger.

Because of a lack of data diversity in the study sample (i.e., consistently high scores across all rounds), the relationship between RDT availability and requesting a malaria test could not be assessed. However, there were two significant findings: when RDT commodities were available, requesting a malaria test increased by a factor of 16 in Cameroon for round 4 and increased by a factor of 42 in Mali for round 3. Similarly, there was a lack of data diversity in the study sample for assessing the relationship between ACT availability and health workers providing the correct treatment to patients.

## DISCUSSION

This study is one of the first to assess the association between facility readiness factors and competency in malaria case management at the country level, specifically in high-burden settings.^5^ Our study’s findings build upon those from MalariaCare and reinforce further the evidence supporting the importance of facility readiness for high-quality malaria case management services.

The analysis of the three rounds of OTSS visits in PMI Impact Malaria–targeted facilities shows that greater facility readiness, represented by critical inputs such as commodities, materials, and documentation, as well as ≥50% of personnel classroom-trained in malaria case management, is associated with greater competency in health workers, providing evidence that facility readiness likely has an effect on providers’ performance. Not surprisingly, health workers who are trained and provided with the necessary materials and commodities are more likely adhere to malaria case management guidelines.

Specific readiness elements may have had more of an effect on health worker competency than others. Significant associations were found between the availability of materials and the competency of health workers in the overall management of patients suspected of having malaria. Although this association was not established in all three rounds in Cameroon and Niger, and was not significant in any OTSS round in Mali, it demonstrates the unique role that the availability of materials can have on the quality of care. Similarly, in Mali, classroom training was associated with improvement of the competency of health workers in managing suspected malaria patients across all OTSS rounds. However, this association was not significant in Cameroon and Niger and could be an example of how different facility readiness factors have a stronger influence in different country contexts.

There was limited evidence of the correlation between the availability of RDTs and ACTs, and health workers requesting malaria testing and prescribing treatment correctly. In Cameroon, there was a strong association in the fourth OTSS round between availability of RDTs and malaria testing. Similarly, in the third round in Mali, a strong association was noted between the availability of RDTs and the competency of health workers to request a malaria test. These associations could not be assessed in other rounds because almost all facilities were well stocked with RDTs and ACTs. In addition, most health workers adhered to the norms of requesting a malaria test for patients suspected of having malaria and prescribing ACTs correctly to treat uncomplicated malaria, providing further evidence to existing literature that OTSS likely contributes to improvements in health worker performance and commodity availability.^5^

Our analysis also assessed the impact of overall readiness of facilities on critical steps in the management of patients suspected of having malaria, such as classifying cases correctly, requesting a malaria test, and providing the right treatment to patients with malaria. A strong association was found only in the third round of OTSS in Mali between overall readiness and requests for a malaria test. An association could not be assessed in the other Mali OTSS rounds nor in any of the Cameroon and Niger rounds because a very large proportion of health workers in all three countries observed during these rounds demonstrated high competency in requesting a malaria diagnostic test correctly, classifying cases correctly as uncomplicated or severe, and providing the correct treatment to patients diagnosed with malaria.

There were some limitations to our analysis. In some instances, the sample sizes were too small to run a regression analysis. In others, the lack of diversity of data between variables, particularly when the competency scores were high for some indicators, limited the ability to assess some associations. Artemisinin-based therapy availability was collected as a binary variable and was disaggregated by four age groups. The study team had to calculate manually an estimated aggregated ACT availability variable, which may have introduced some inaccuracies during the aggregation process. The limited sample size also prevented multivariable regression analysis to test for confounding effects and have more conclusive results for some variables, such as the impact of readiness adherence to a negative RDT.

Our study provides further evidence that strengthening and sustaining health facility readiness is an important input toward improving the quality of malaria case management. Based on our results, health facility readiness should be an essential component of a systems-based, integrated, and tailored approach for improving the quality of malaria services in high-burden countries. In addition, the analyses presented herein should be embedded into regular programmatic assessments on quality-of-care trends using well-defined indicators at all levels to allow corrective actions when and where gaps are identified.

## Supplemental Materials

10.4269/ajtmh.23-0479 Supplemental Materials
